# Embedded Processing for Extended Depth of Field Imaging Systems: From Infinite Impulse Response Wiener Filter to Learned Deconvolution

**DOI:** 10.3390/s23239462

**Published:** 2023-11-28

**Authors:** Alice Fontbonne, Pauline Trouvé-Peloux, Frédéric Champagnat, Gabriel Jobert, Guillaume Druart

**Affiliations:** 1 DOTA, ONERA, Université Paris Saclay, 91123 Palaiseau, France; 2 DTIS, ONERA, Université Paris Saclay, 91123 Palaiseau, France; 3 LYNRED, Route de Valence, 38113 Veurey-Voroize, France

**Keywords:** deconvolution, Wiener filter, end-to-end design

## Abstract

Many works in the state of the art are interested in the increase of the camera depth of field (DoF) via the joint optimization of an optical component (typically a phase mask) and a digital processing step with an infinite deconvolution support or a neural network. This can be used either to see sharp objects from a greater distance or to reduce manufacturing costs due to tolerance regarding the sensor position. Here, we study the case of an embedded processing with only one convolution with a finite kernel size. The finite impulse response (FIR) filter coefficients are learned or computed based on a Wiener filter paradigm. It involves an optical model typical of codesigned systems for DoF extension and a scene power spectral density, which is either learned or modeled. We compare different FIR filters and present a method for dimensioning their sizes prior to a joint optimization. We also show that, among the filters compared, the learning approach enables an easy adaptation to a database, but the other approaches are equally robust.

## 1. Introduction

Optical systems naturally have a limited depth of field (DoF), and it is a common problem to wish to increase it. This can be used either to see sharp objects from a greater distance or to reduce manufacturing costs due to tolerance regarding the sensor position. One way of doing this is to use a joint optical/digital design technique (or “co-design”). In this context, the variation of the optical system’s point spread function (PSF) as a function of the depth of field is reduced to the detriment of the image quality of the sensor. The use of a cubic phase mask is a typical way of achieving this PSF invariance [1]. Digital processing is then required for restoration [2] and (preferably) optimized in conjunction with the optical system. This means that digital processing parameters depend on optical system parameters, but it also means that optical system parameters depend on digital processing. Among the different digital processing techniques, neural networks can be used [3,4,5,6,7,8,9]. However, some applications (e.g., advanced driver assistance systems) require real-time digital processing, with a very limited memory storage ability. These applications are also very limited in terms of power consumption: certain industrial constraints limit camera power consumption (including digital processing) to 1 W. In this case, even approaches such as the use of an infinite impulse response (IIR), Wiener filter, or mixed deep learning/Wiener approaches [10,11,12] are no longer usable. For these applications, it is only possible to implement a linear filter with a finite impulse response (FIR) [13,14,15]. Indeed, current industrial constraints for low-consumption, embedded real-time systems require the use of FPGA boards [16,17,18]. Storage (for example, for recording a deconvolution kernel) is limited, and operations have to be performed as the image is acquired (at almost every line); otherwise, the entire image would have to be stored. This drastically limits the weight of the restoration algorithm that can be used. Yet, in an end-to-end, optical/digital design context for DoF extension, the PSFs of the optical system are often large [1,19,20], which can be problematic if one wishes to deconvolve with a FIR filter, especially with a very small kernel.

**Figure 1:**
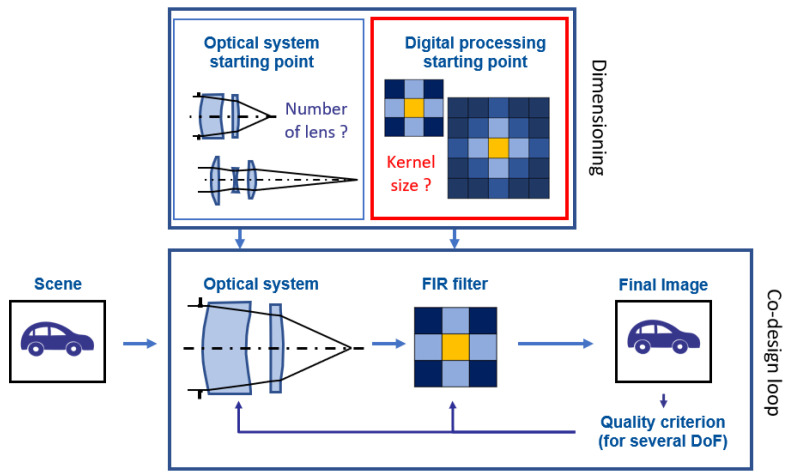
A classical codesign loop iterates on optical system and filter parameters in order to optimize a merit function. The initial state is provided by the dimensioning task. The red box corresponds to the part studied in this article.

In this article, we focus on a step preceding the co-optimization of a hybrid optical/digital system (Figure 1). One of these first steps is to dimension the real-time digital processing to be applied and co-optimized with the optical part (i.e., choose its preliminary specifications) in order to enhance the DoF [21]. The size of the deconvolution kernel is one of the variables to be chosen before codesign, in the same way as the number of lenses, the number of active surfaces, and the surface parameterization (aspherization, freeform, etc.) of an optical system are chosen before optimization begins. The aim of this article is twofold. The first is to propose a methodology for choosing the size of the deconvolution kernel. The second is to evaluate the potential impact of this choice on the result after the co-optimization of the imaging system. Our contributions are the following. We build a simple performance model that predicts the mean square error (MSE) after deconvolution. Based on this performance model, we quantitatively compare the improvement, in equivalent settings, provided by different FIR filter types: either a truncation of the IIR Wiener Filter, a FIR Wiener filter, or a FIR filter with learned coefficients. Furthermore, we evaluate the benefit of the learning approach compared to a model-based approach. In this article, we first describe in Section 2 the pipeline of the imaging system consisting of an optical system and different possible deconvolution filters. Then, in Section 3, we study the effect of different kernel sizes as a function of the PSF width and noise. This provides a method for determining an appropriate deconvolution kernel size. At the end of this results section, we focus on the contribution of learning the FIR filter coefficients for deconvolution on real images. Finally, we conclude on the evolution of the image quality as a function of the deconvolution kernel size for the different studied FIR filters.

## 2. Description of the Processing Pipeline

In this article, we consider a usual image restoration model I(r)=w★(h★O+n)(r), where *r* values are the spatial (2D) coordinates, w(r) is the deconvolution filter, h(r) is the PSF, and ★ stands for the convolution operation. O(r) is the perfect scene image, which is assumed to be a zero-mean and unitary variance, as well as a stationary random process of power spectral density (PSD) $S_{OO}\left(\nu \right)$ (with ν being the spatial frequency coordinates, which are normalized to the Nyquist frequency of the sensor). The noise n(r) is modelled as a zero-mean Gaussian distribution. Its standard deviation depends on the chosen signal to noise ratio (SNR). Its (constant) PSD is denoted as $S_{nn}\left(\nu \right)$. We measure the SNR in dB according to $SNR=10\log_{10}\frac{\int S_{OO}\left(\nu \right)d\nu }{\int S_{nn}\left(\nu \right)d\nu }$.

### 2.1. PSD Model and Image Database

**Figure 2:**
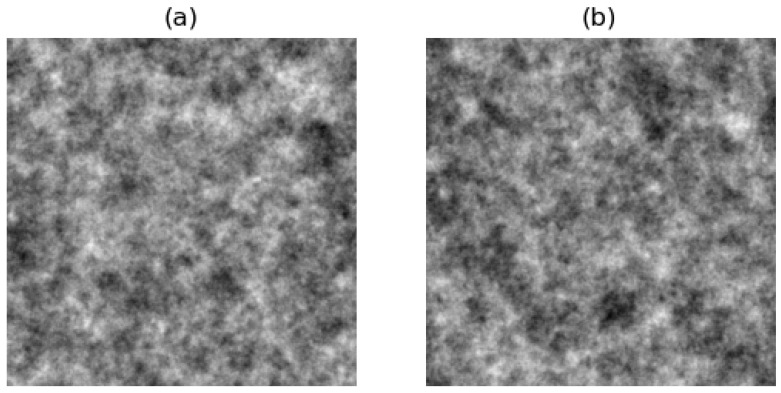
(**a**) An image generated from the Reichenbach PSD model. (**b**) Another image generated from the same model.

**Figure 3:**
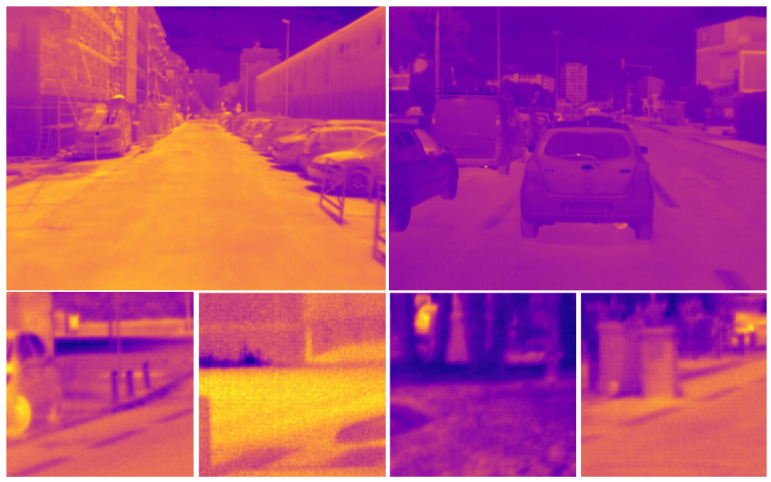
First row: two random full images from the database. Second row: four random patches from the database.

Estimating the O(r) from I(r) is an ill-posed problem that requires the use of a priori knowledge about the O(r), either via a model or a database. Both must be representative of the observed scene. We consider the following “Reichenbach PSD model” from [22]:

(1) $$S_{OO}\left(\nu \right)=\frac{2\pi \mu^{2}}{{\left(1+4\pi^{2}\mu^{2}\nu^{2}\right)}^{3/2}}\;\;\;\;,$$

with 1/μ being homogeneous to a cutoff frequency and $\int S_{OO}\left(\nu \right)d\nu$ being normalized to one. μ is related to the mean spatial detail. The value of μ has been set to 15 in order to be in rough agreement with the PSD of the real scenes database described hereafter. In this paper, we consider two databases: one generated from a Reichenbach model described in Section 3 (Section 3.1, Section 3.2 and Section 3.3) and the other from an automotive database of real long-wave infrared images [23] described in Section 3.4. The first is directly generated using the definition of the power spectral density, which is multiplied in the Fourier domain to become a matrix of randomly drawn numbers. This results in images that are not visually representative of real scenes (Figure 2) but have a controlled, realistic spectral content. The second is composed of 5943 images of size 640×480. It has been captured while driving in an urban environment (with pedestrians and cars at a distance of about 1 to 30 m) both during the day and at night. Examples of the images from this database are given in Figure 3.

### 2.2. PSF Model for System with Extended DoF

In this article, we aim to dimension a system that will be codesigned as described in Figure 1. In particular, this dimensioning concerns the choice of the deconvolution filter size and shape, which depend a priori on the size of the kernel to be deconvolved (the PSF). However, the PSF is unknown, as the optical system is still at the dimensioning stage. Therefore, a model is needed to predict the general shape of the PSF and its spread over the sensor. Here, the model is established from previous codesign results based on the use of phase masks in the pupil of a system at the diffraction limit, such as cubic phase masks, annular binary phase masks, or other shapes [24]. These systems, with their extended DoF values, have the particularity of presenting very specific modulation transfer functions (MTFs) with a sharp drop-off at low spatial frequencies and signal conservation at medium and high spatial frequencies [25].

**Figure 4:**
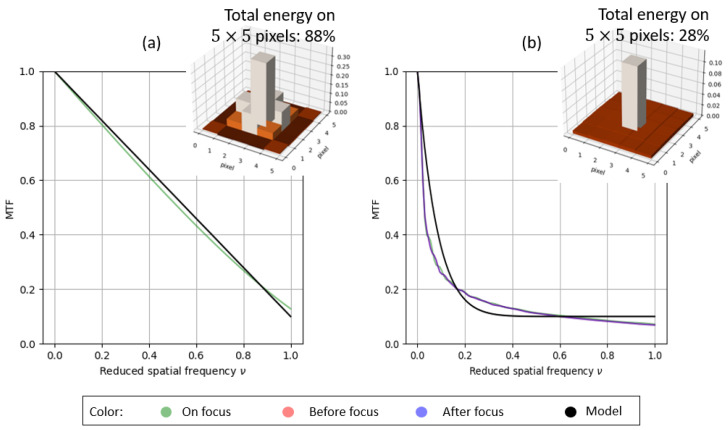
(**a**) ${\tilde{h}}_{1,0.9}\left(\nu \right)$ with regard to the MTF of a diffraction-limited system (with low DoF) and the corresponding PSF with a 5×5 support size. (**b**) ${\tilde{h}}_{12,0.9}\left(\nu \right)$ with regard to the MTFs of an extended DoF system with a cubic phase mask. “Before focus” and “After focus” curves are superimposed.

We propose to account for this behavior with the following model for all the MTFs through their DoF values:

(2) $${\tilde{h}}_{\rho ,a}\left(\nu \right)=a\times {\left(1-|\nu |\right)}^{\rho }+\left(1-a\right)\;\;\;\;,$$

with $\tilde{\cdot }$ denoting the Fourier transform and *a* and ρ denoting the two parameters of the model. (1−a) is the value of the MTFs at the normalized Nyquist frequency. ρ is a parameter related to the expected DoF extension: ${\tilde{h}}_{1,a}\left(\nu \right)$ models a kind of diffraction-limited optical system (with a low DoF) as it becomes an affine function. An example is given in Figure 4a. The larger is ρ, the faster the drop in the MTF. It enables us to reach an enhanced DoF. Figure 4b shows the example of a system with a cubic phase mask [1], thereby aiming to greatly extend the DoF.

**Figure 5:**
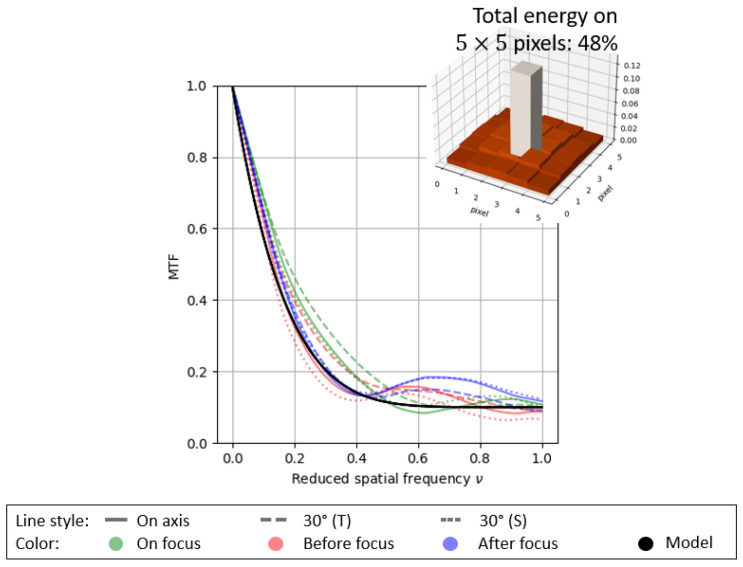
${\tilde{h}}_{6,0.9}\left(\nu \right)$ with regard to the MTF of an extended DoF–F/0.8 system (with phase mask derived from [2]) and the corresponding PSF with large base centered on a 5×5 support size. “Before focus” and “After focus” are in the DoF extension optimized range of the phase mask.

To give another example of the model’s reliability, Figure 5 shows the MTFs of an extended DoF–F/0.8 system (with a phase mask derived from [2]), which is invariant with respect to the field of view and DoF. Note that this system on an axis and without a phase mask is diffraction-limited, given an MTF at 0.4 in the Nyquist frequency. In this latter case and with a binary phase mask, we also observe a sharp drop for the low frequencies and a preservation of the high frequencies (with no nullings), which are characteristics of codesigned systems. This drop in the MTFs for low frequencies naturally generates a wider base for the PSF compared to a diffraction-limited optical system. Therefore, the PSF energy is less concentrated around the origin for a codesigned system (Figure 4 and Figure 5).

In the rest of the article, we set the parameter *a* to 0.9, thus considering that if the MTF were to take values below 0.1, it would be below the noise levels for most realistic applications [25].

### 2.3. The MSE Criterion

In this article and otherwise stated, the MSE (between the ideal image of the scene and the final image [25]) is the optimization and evaluation criterion before and after processing. Any evaluation of “the best filter” is therefore made in the sense of the MSE. It is computed by its analytical formula in Section 3 and empirically for the images at the end of this results section.

### 2.4. Different FIR Filters

To restore an image with a linear deconvolution filter, the best filter is the IIR Wiener filter (which requires the *a priori* knowledge of $S_{OO}\left(\nu \right)$ for its construction):

(3) $$\tilde{w}\left(\nu \right)=\frac{\tilde{h^{*}}\left(\nu \right)}{{|\tilde{h}\left(\nu \right)|}^{2}+\frac{S_{nn}\left(\nu \right)}{S_{OO}\left(\nu \right)}}\;\;\;\;,$$

with $\tilde{h^{*}}\left(\nu \right)$ being the complex conjugate of $\tilde{h}\left(\nu \right)$. It gives the best achievable MSE with a linear filter, but this filter has an infinite support, which makes it unusable with the hardware constraints considered in this article. In addition to this filter, we are interested in three types of FIR filters, which respect the hardware constraints. The three filters are built knowing the size K×K of the filter (*K* is odd), the model of the PSF, and the SNR:

- The simplest approach is to truncate the IIR Wiener filter, in the direct space, to the chosen kernel size K×K. It will be noted as the “Truncated IIR Wiener filter” in the following. Since we consider small kernel sizes, we use a simple rectangular windowing and no apodization, which is in contrast with [26,27,28,29].
- A second approach consists of optimizing a “FIR Wiener filter”, i.e., finding the best linear filter under the constraint of a finite kernel size [22]. It needs the autocorrelation of the scene for its construction, which is calculated from the scene PSD using a Fourier transform.
- Finally, the “learned filter” corresponds to the minimization over the K×K filter coefficients of the MSE criterion averaged on an image database (which plays the role of the scene PSD). We use the Adam optimizer for that, with a batch size of 10 and 0.01 as the learning rate. The choice of starting point will be discussed in Section 3.

## 3. Results

In this section, we compare the deconvolution performance outcomes of different FIR filters with respect to various cases. The scene was first represented by the Reichenbach model, from which we generated a training dataset for learning.

### 3.1. First Case Study

**Figure 6:**
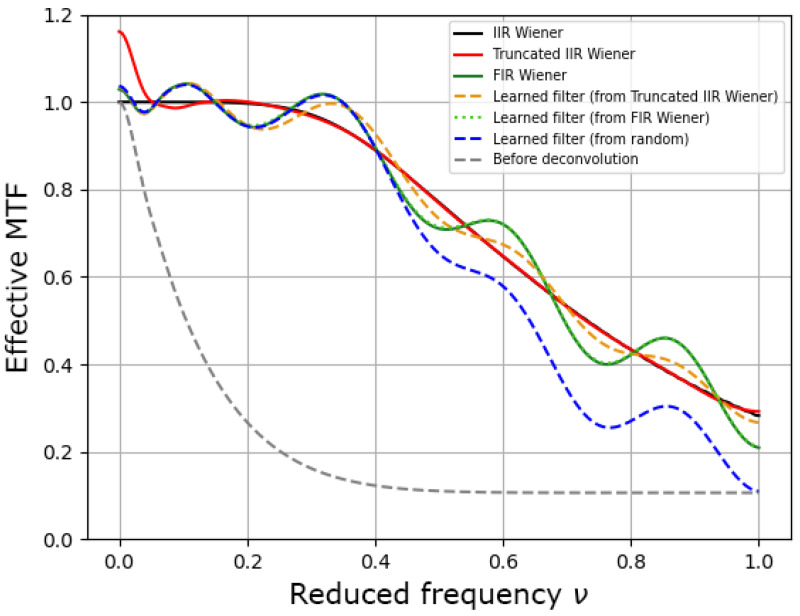
Effective MTFs calculated from the different IIR and FIR filters limited to a 17×17 kernel.

We compared the three types of filters presented in Section 2 in a first case where the deconvolution kernel remained large (17×17) and with ρ=8 and SNR=34 dB. We first compared the different effective MTFs (defined by $|\tilde{h}\left(\nu \right)\times \tilde{w}\left(\nu \right)|$), i.e., the transfer functions after deconvolution, which are shown in Figure 6. Both the Truncated IIR Wiener filter and the FIR Wiener filter were close to the IIR Wiener filter, but the first one clearly modified the mean of the image, while the second oscillated around the best effective MTF. We tested several starting points for the learning approach: either starting from random coefficients, from the Truncated IIR filter, or directly from the FIR filter. The learned filter, when starting from the FIR Wiener filter, showed no change in the effective MTF. Indeed, the FIR Wiener filter already corresponded to the theoretical optimal filter. Starting the learning from the random or Truncated IIR Wiener filter converged to solutions that were close to the FIR Wiener filter, in particular for the lowest frequencies, but that were differ slightly for the highest ones. The optimization was also much longer when the starting point was random. Reaching the theoretical optimal solution using learning requires a careful choice of the starting point and of the learning hyperparameters (e.g., by increasing the batch size and using another optimizer specifically for this problem, such as via LBFGS).

In terms of the MSE (Table 1), the filters learned from the FIR Wiener filter and from the Truncated IIR Wiener filter yielded equivalent results that were close to the optimal one (the IIR Wiener filter). Hence, in the following, we systematically considered the Truncated IIR Wiener filter as the starting point for learning: this filter is close to the IIR Wiener filter, except for at very low spatial frequencies (Figure 6).

### 3.2. Impact of Deconvolution Kernel Size on Image Quality

**Figure 7:**
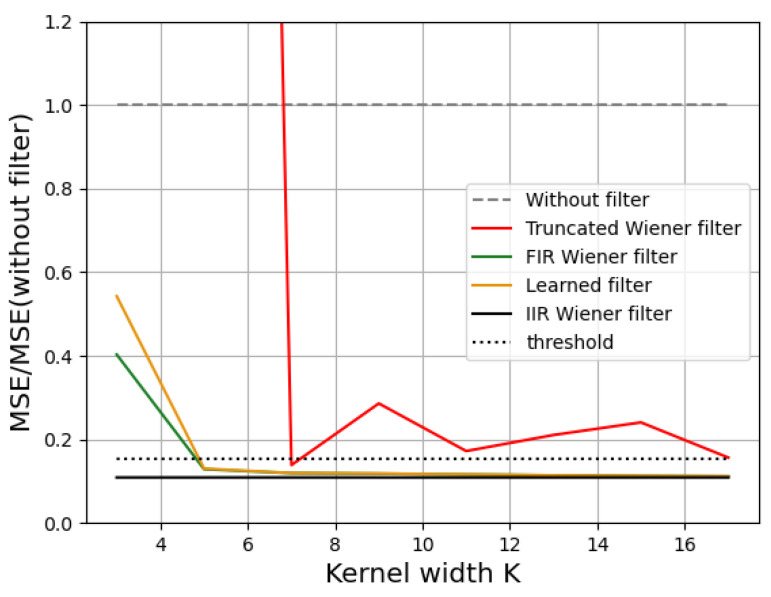
Theoretical evolution of the MSE with different filters as a function of the deconvolution kernel size in the case of a Reichenbach PSD, with ρ=8 and SNR=34 dB.

We expanded the previous study to different kernel sizes while still having ρ=8 and SNR=34 dB. Figure 7 shows the variation in the MSE with respect to the kernel width *K* for the three methods. The MSE globally decreased when *K* increased. This is an expected result for the FIR filter [22], and Figure 7 confirms that, even though the learned filter had a different shape (Figure 6), it yielded similar results to the best (FIR) filter. The Truncated IIR Wiener filter yielded the worse MSE performance, especially for very small kernels. We noticed an asymptotic behavior, with no need to use a large kernel to have a good reconstruction. For this case study, K=5 was sufficient to achieve 95% of the best result with a linear filter, which was obtained with the IIR Wiener filter. This 95% threshold is plotted as shown by the dotted line in Figure 7.

### 3.3. Method for Choosing the Kernel Size

**Figure 8:**
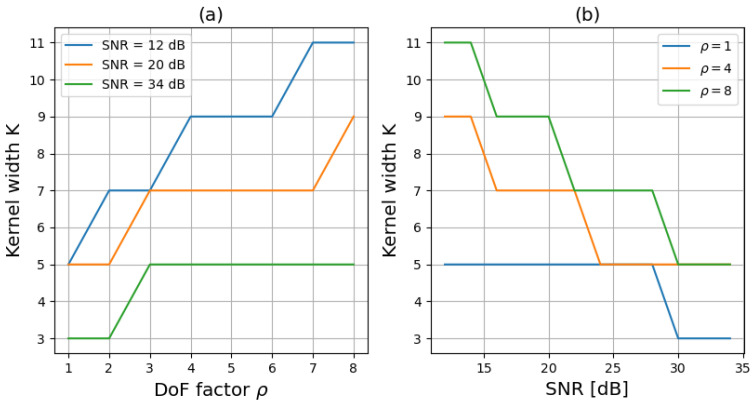
(**a**) Size of the kernel depending on ρ (**b**) Size of the kernel depending on SNR to reach the 95% threshold.

We reproduced this study for different ρ values, different SNR values, and different kernel sizes. With a similar 95% threshold as in Figure 7, we could deduce the minimal size of the deconvolution kernel to obtain nearly the best image quality with a linear filter (Figure 8). This minimal size was obtained with the FIR Wiener filter, as well as with the learned filter. With respect to increasing the DoF (with increasing ρ), the more abruptly the MTF dropped for low frequencies, the larger the base of the PSF was, and the larger the kernel of the deconvolution filter needed to be. Furthermore, a larger *K* was needed for low SNR values. Indeed, when the noise is very important, the reconstruction is done mainly for the low spatial frequencies. The deconvolution filter (in the Fourier space) is therefore tightened even if the PSF is large. With such an approach, a deconvolution filter of size 5×5 appears sufficient when the thermal contrast is good (34 dB), even in the context of increasing the DoF by codesign. It will however be necessary to increase this size for lower SNR values. For example, a size of 9×9 is preferable at 20 dB.

### 3.4. Robustness on Real Images

**Figure 9:**
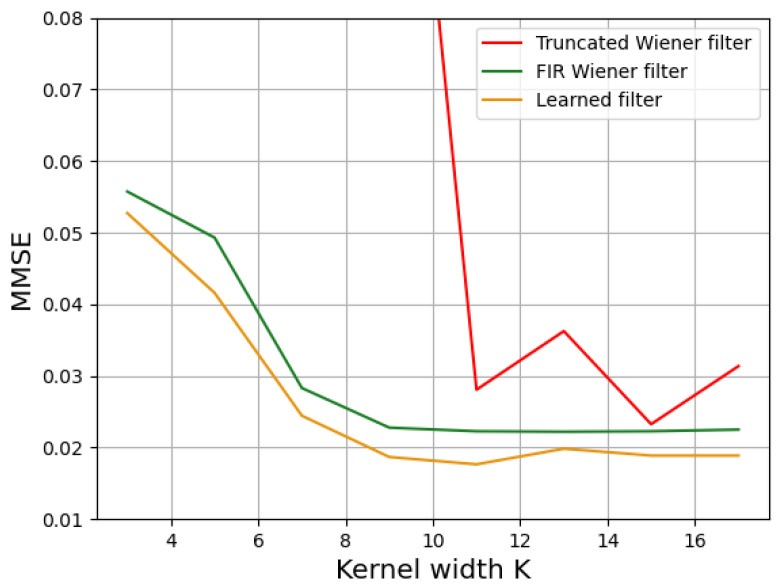
Variation in the MMSE for the test dataset with respect to the deconvolution kernel size with respect to the automotive database.

**Figure 10:**
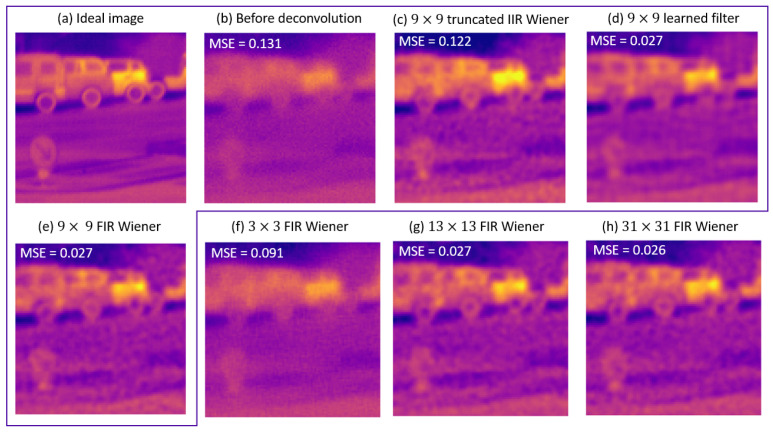
Patch of an image of the database, with ρ=8 and SNR=20 dB. (**a**) Ideal image. (**b**) Simulated acquired image. Deconvolution with a 9×9 kernel size. (**c**) Truncated Wiener filter. (**d**) Learned filter. (**e**) FIR Wiener filter. (**f**–**h**): other FIR filter sizes.

We now consider the case where the true scene statistics differs substantially from the Reichenbach model. Learning makes it possible to take the differences between a real scene and an analytic model into account. We consider here an automotive database. Over the entire database, we can calculate an average MSE (MMSE) that is equivalent to the theoretical MSE calculated above. As shown in Figure 9, we obtained an evolution similar to the one studied in Section 3; here, this was obtained with ρ=8 and SNR=20 dB, while still considering the normalized Reichenbach PSD for Wiener filters. The Truncated IIR Wiener filter needed a large kernel size to be efficient, while the FIR Wiener filter quickly yielded asymptotic results. Learning this allowed us to improve the performance outcomes. The associated MMSE curve followed that of the Wiener FIR filter but at a lower level: the learning process allowed us to adapt the filtering to the new PSD. Indeed, the FIR Wiener filter was no longer optimal, because the model used to define the filter was less representative of the database. Nevertheless, it remained relatively robust. The results can be seen in Figure 10. In this figure, we are interested in a subsection of an image from the database used. The various reconstructions were compared with the ground truth (or “ideal image”, which is shown in Figure 10a) and the simulation of the passage of this image through the optical system (taking noise into account, which is shown in Figure 10b). Qualitatively, the reconstruction with the FIR filter seems to give the most details. However, the noise was also amplified and yielded an orange peel effect (Figure 10e). This effect is visible regardless of the size of the deconvolution filter (Figure 10e–h). The learned filter made a better compromise in the sense of the MSE (Figure 10d): the image is less sharp, but the noise is less amplified. Note that similar results can be observed with the other images in the database and also with other databases [30].

For the case of the 9×9 kernel, the Truncated Wiener filter yielded good visual results despite a strong MSE. This was not the case when the kernel was very small (<7×7), but, when the kernel was 9×9 in size (or larger, see Figure 6), this result was expected. Indeed, according to the effective MTFs shown in Figure 6, mainly the very low frequencies were not well reconstructed: the mean value of the reconstructed image is wrong, but this has little visual impact. We quantified the impact of processing on the database using two typical and complementary image processing metrics: the MMSE and the structural similarity index measure (SSIM) [31]. As shown in Table 2, this bad reconstruction of the mean had an impact on both the MSE and the SSIM, but this impact was lower for the second metric.

Therefore, this learning approach allowed for an improvement in the MSE by better taking into account the true PSD of the scene, whereas the Wiener approaches used a template PSD. Even if the MSE was the optimization loss, the obtained filter was also better on the SSIM. The Wiener FIR filter still offered good visual performance outcomes, even for very small deconvolution kernel sizes.

## 4. Conclusions

In this article, we have used FIR filters with codesigned systems. In particular, we have compared FIR filters for the image restoration of an optical system with an extended DoF (i.e., with a large base PSF). For the learning approach, we have shown the interest of initializing the learning with a truncated Wiener filter, both from the point of view of the learning time and the result found. Furthermore, we have established a method to determine a minimum kernel size as a function of a model of the noise and the MTFs of an extended DoF system based on the Wiener FIR filter results. We found that, depending on the noise level, a filter size of 5×5 to 9×9 could be sufficient for most DoF extensions using a Wiener FIR filter or a learned filter. This is a very important step in the design of a hybrid optical digital system. Finally, we have seen that the learning process can further improve the results through small adaptations to the database characteristics, but these improvements remain small. Indeed, this improvement is mainly for the high spatial frequencies whose restoration depends on the noise level.

The perspectives of this work are numerous. First, the actual embedding of the deconvolution filter requires the quantification of the coefficients of the deconvolution kernel, and this involves a study of the impact of this quantification on the image quality. This quantification could further limit the MSE, and this is the main limitation to this work. The second, and most important one, is to achieve the end-to-end optimization of an imaging system (optics and processing) starting from a well-chosen starting point: it is now possible to choose a kernel size that is well adapted to each DoF extension problem. It could also allow us to use metrics that are closer to visual perception. In addition, it will be possible to consider a live adaptability with several operating modes depending on the SNR.

## Figures and Tables

**Table 1:** First row: MSEs of the different filters. Second row: MSEs of the filters learned from first row.

	IIR Wiener	Truncated IIR Wiener	FIR Wiener	Random
Before learning	0.0146	0.0210	0.0150	-
After learning	-	0.0150	0.0150	0.0160

**Table 2:** Average values of MMSE and SSIM for patches of automotive database for deconvolution with different 9×9 filters, with ρ=8 and SNR=20 dB.

	No Filter	Truncated IIR Wiener	FIR Wiener	Learned Filter
MMSE	0.0642	0.1591	0.0234	0.0177
SSIM	0.3984	0.4825	0.5253	0.6039

## Data Availability

The code underlying the results presented in this paper is not publicly available at this time but may be obtained from the authors upon reasonable request. The automotive dataset is not publicly available.

## Supplementary Materials

The following supporting information can be downloaded at: https://www.mdpi.com/article/10.3390/s23239462/s1: Figure S1: PSD as a function of the (normalized) spatial frequency of two scenes of the Infrared database and modeled by the Reichenbach model with μ=15.; Figure S2: (a,b) Sample 2D PSFs corresponding of those of Figure 4 of the article (with a logarithmic scale).; Figure S3: Modulus of filters used in Figure 6. (a) IIR Wiener Filter (b) Truncated IIR Wiener filter (c) FIR Wiener filter (d) Learned filter (from random) (e) Learned filter (from Truncated IIR Wiener filter) (f) Learned filter (from FIR Wiener filter); Figure S4: Cross-section of the different IIR and FIR filters, limited to a 17×17 kernel.; Figure S5: Filters used in Section 3, for deconvolution of images (Figure 10). (a) Truncated IIR Wiener filter (b) FIR Wiener filter (c) Learned filter; Figure S6: First row: Ideal image (patch) or “ground truth”. Second row: Simulation through the optical system, with 20 dB SNR and deconvolution with the 9×9 FIR filter. (a,b) A random patch of the database; (c,d) Another random patch; (e,f) Another random patch; (g,h) Another random patch.

## Abbreviations

The following abbreviations are used in this manuscript:

DoF	Depth of Field
PSF	Point Spread Function
IIR	Infinite Impulse Response
FIR	Finite Impulse Response
SNR	Signal-to-Noise Ratio
MTF	Modulation Transfer Function
MSE	Mean Square Error
PSD	Power Spectral Density
