# Supplementary material for: Embedded Processing for Extended Depth of Field Imaging Systems: From Infinite Impulse Response Wiener Filter to Learned Deconvolution
Source: Sensors (Basel). 2023 Nov 28;23(23):9462. doi: 10.3390/s23239462 (PMC10708841; doi:10.3390/s23239462)
Supplement: Supplementary file 1 [file sensors-23-09462-s001.zip › sensors-2682453-supplementary.pdf]

# Supplementary Material

"Embedded processing for extended depth of field imaging systems: from Infinite Impulse Response Wiener Filter to Learned Deconvolution"

Alice Fontbonne, Pauline Trouvé-Peloux,
Frédéric Champagnat, Gabriel Jobert, Guillaume Druart

In this document, we present a few additional figures to complement the article for the interested reader.

## 1 About the Reichenbach's model

In the article, we wrote that "the value of $\mu$ [of the Reichenbach's Power Spectral Density (PSD) model] has been set to 15 in order to be in rough agreement with PSD of the real scenes database described hereafter." We have checked the compatibility of this value with some PSD of images in the database. An example is shown in Figure S1.

## 2 About the optical model

In the article, we have described a Point Spread Function (PSF) model. Two of them are shown in Figure 4. We show on Figure S2 the PSFs corresponding to Figure 4 on $60 \times 60$ pixels.

## 3 About deconvolution filters of the first case study

A representation of the used deconvolution filters in Fourier space may be of interest. We take here the example of Figure 6 from the first case study (with a filterof size $17 \times 17$). Figure S3 is presented the corresponding deconvolution filter modulus, in 2D.

As they are almost rotationally symmetrical (due to the definition of PSD and PSF), a cross-section is also appropriate to compare the filters with each other. This representation is given on Figure S4.

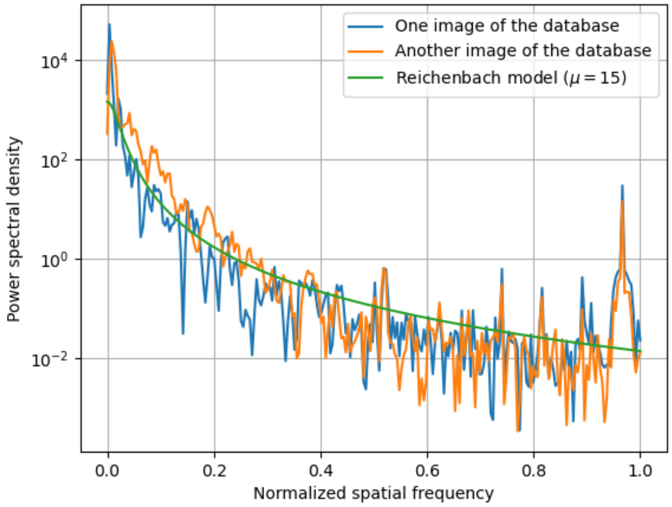


Figure S1: PSD as a function of the (normalized) spatial frequency of two scenes of the Infrared database and modeled by the Reichenbach model with $\mu = 15$.

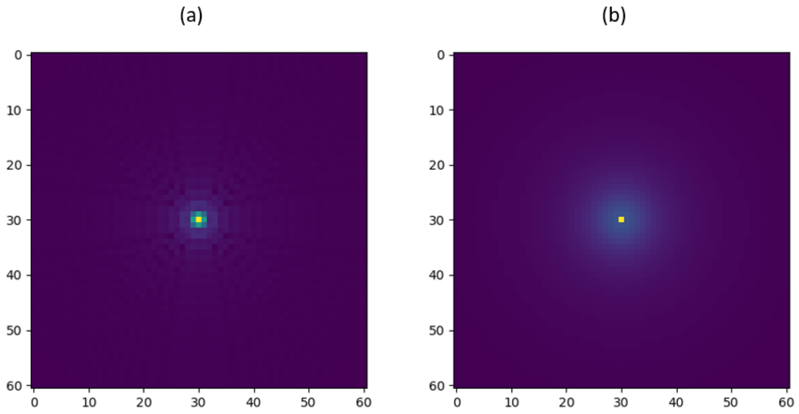


Figure S2: (a,b) Sample 2D PSFs corresponding of those of Figure 4 of the article (with a logarithmic scale).

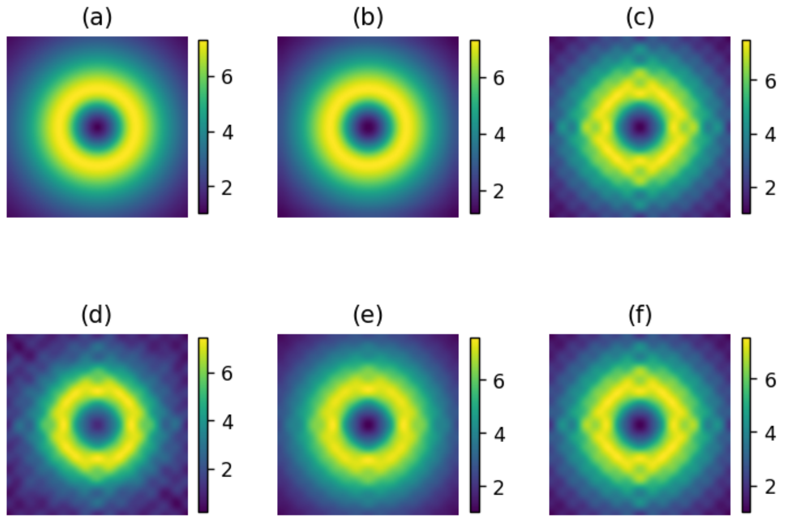


Figure S3: Modulus of filters used in **Figure** 6. (a) IIR Wiener Filter (b) Truncated IIR Wiener filter (c) FIR Wiener filter (d) Learned filter (from random) (e) Learned filter (from Trun-cated IIR Wiener filter) (f) Learned filter (from FIR Wiener filter).

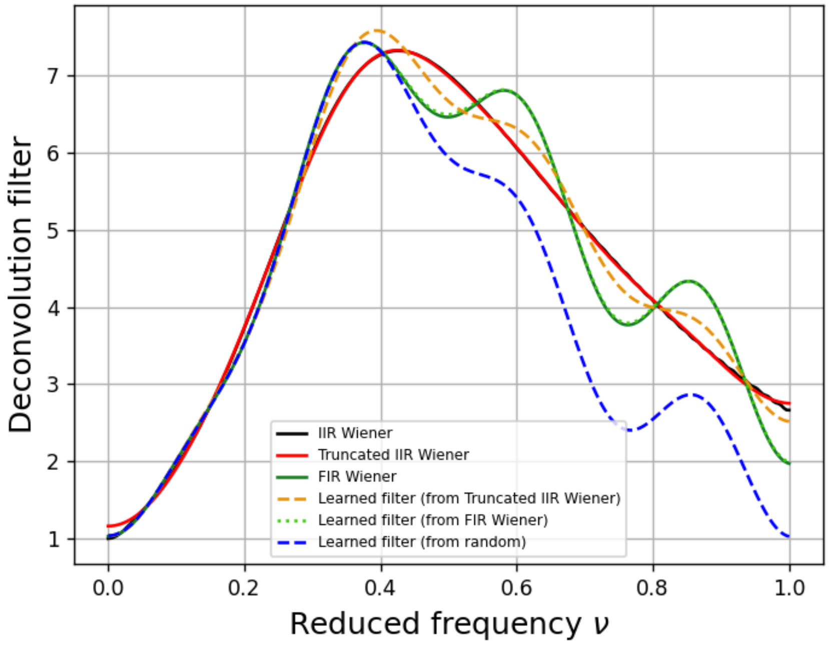


Figure S4: Cross-section of the different IIR and FIR filters, limited to a $17 \times 17$ kernel.

# 4 About image simulation

Figure S5 shows the $9 \times 9$ filters used in section 3 of the article for image simulation. The impact of these filters can be seen directly on the image simulations in Figure 10.

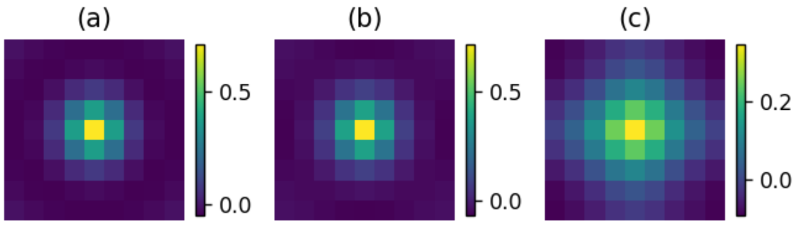


Figure S5: Filters used in section 3, for deconvolution of images (Figure 10). (a) Truncated IIR Wiener filter (b) FIR Wiener filter (c) Learned filter.

To complete Figure 10, here are on Figure S6 some simulations on random patches of the database.

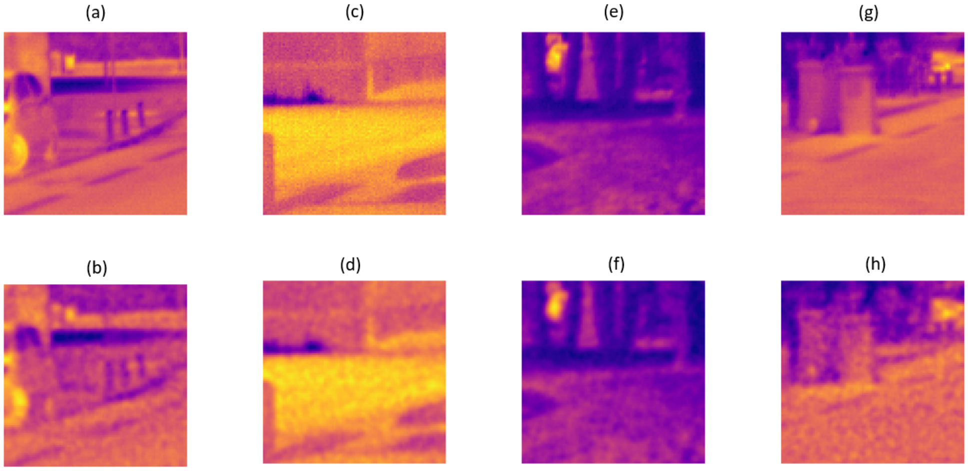


Figure S6: First row: Ideal image (patch) or "ground truth". Second row: Simulation through the optical system, with 20 dB SNR and deconvolution with the $9 \times 9$ FIR filter. (a-b) A random patch of the database; (c-d) Another random patch; (e-f) Another random patch;(g-h) Another random patch.
